# Supplementary material for: L-Rhamnose Globally Changes the Transcriptome of Planktonic and Biofilm Escherichia coli Cells and Modulates Biofilm Growth
Source: Microorganisms. 2024 Sep 19;12(9):1911. doi: 10.3390/microorganisms12091911 (PMC11434101; doi:10.3390/microorganisms12091911)
Supplement: Supplementary file 1 [file microorganisms-12-01911-s001.zip › Supplemental for Zip_postrev/RhamnoseBiofilmSupplemental_FINAL_postrev2.pdf]

Supporting Information

L-rhamnose globally changes the transcriptome of planktonic and biofilm *Escherichia coli* cells and modulates biofilm growth.

Charlotte Hantus[§], Isabella Moppel[§], Jenna K. Frizzell, Anna Francis, Kyogo Nagashima, Lisa M. Ryno*

[§]These authors contributed to this work equally
[*] To whom correspondence should be addressed:
e-mail: lryno@oberlin.edu
Telephone: 440-775-8238
Facsimile: 440-775-6682.

Table of Contents

Page

Table S1: S3

Figure S1: S4

Figure S2: S5

Figure S3: S6

Figure S4: S7

Figure S5: S8

Figure S6: S9

Figure S7: S10

Table S2 .... Separate File

Table S3 .... Separate File

Table S4 .... Separate File

Table S5 .... Separate File

Table S1. qPCR primer sequences.

Gene	Forward Primer Sequence (5'-3')	Reverse Primer Sequence (5'-3')	Reference
bhsA	TAAGCTCCATGTCATTTGCC	TTCCATGGAGGGTATTCGG	68
bscA	AACCACCGGGTCAGGATAC	GGACGCGAGGCGTTGATATG	this study
crp	CCGTCAGGAAATTGGTCAGA	TGCGTCCCACGGTTTCA	81
csgA	GCGGTAATGGTGCAGATGTTG	GAAGCCACGTTGGGTCAGA	68
fimA	TGCTGTCGGTTTTAACATTC	ACCAACGTTTGTTGCGCTAC	82
lptA	CGGCGAACAAGGTAAAGAAG	TTGCCAGTTCGTAGTGCATC	83
mcbA	GCTGACCGCTGTCCAGGTTA	CGTCCTGAGCGGAATCTCTCT	68
mlc	GTCAGCACATCAGCGTTGAGA	CCTGCAACAGACGAATCAACA	84
rhaT	GGTGATGTGCGGCATTTTCT	ACAGTGGATCGACGCCAAGT	84
rrsA	CTCTTGCCATCGGATGTGCCCA	CCAGTGTGGCTGGTCATCCTCTCA	63
sfsA	CAGCGTGCGGTTATCTTTTTC	CTACCCCCCTCTGTTGAGCTT	84
wcaF	TCTCGGTGCCGAAAGGGTTC	ATTGACGTCATCGCCGACCC	82
xylF	CTGCACACGCCAAAGAAGTC	GTGTTTCTTCATTGCCATTTGC	84

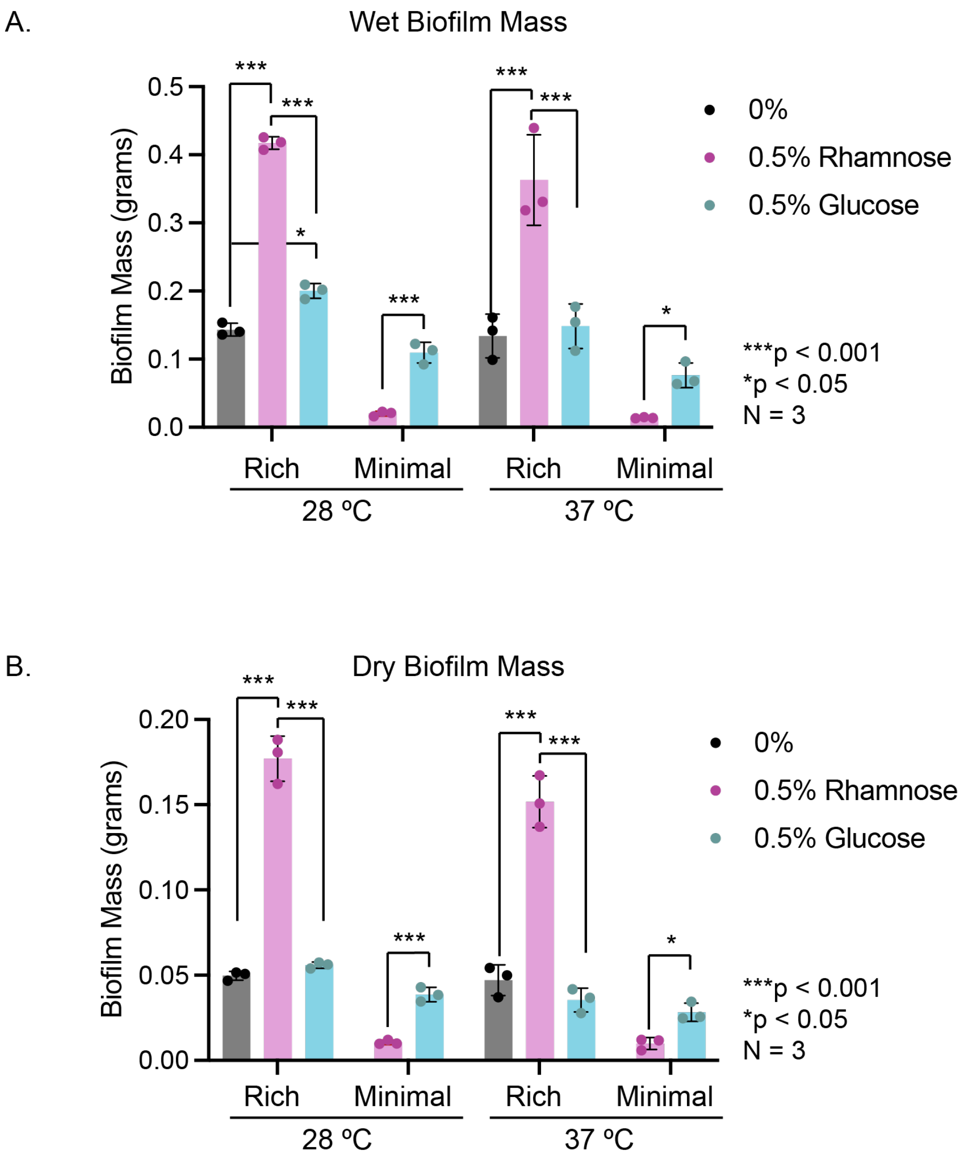

Figure S1. Mass measurements of **A.** wet and **B.** dry biofilm from agar plate growth. Biofilm was grown on LB (rich) or M9 (minimal) agar plates supplemented with either no added sugar (0%) 0.5% (*w/w*) rhamnose or 0.5% (*w/w*) glucose. Statistics were determined using a two-way ANOVA with post-hoc Tukey test.

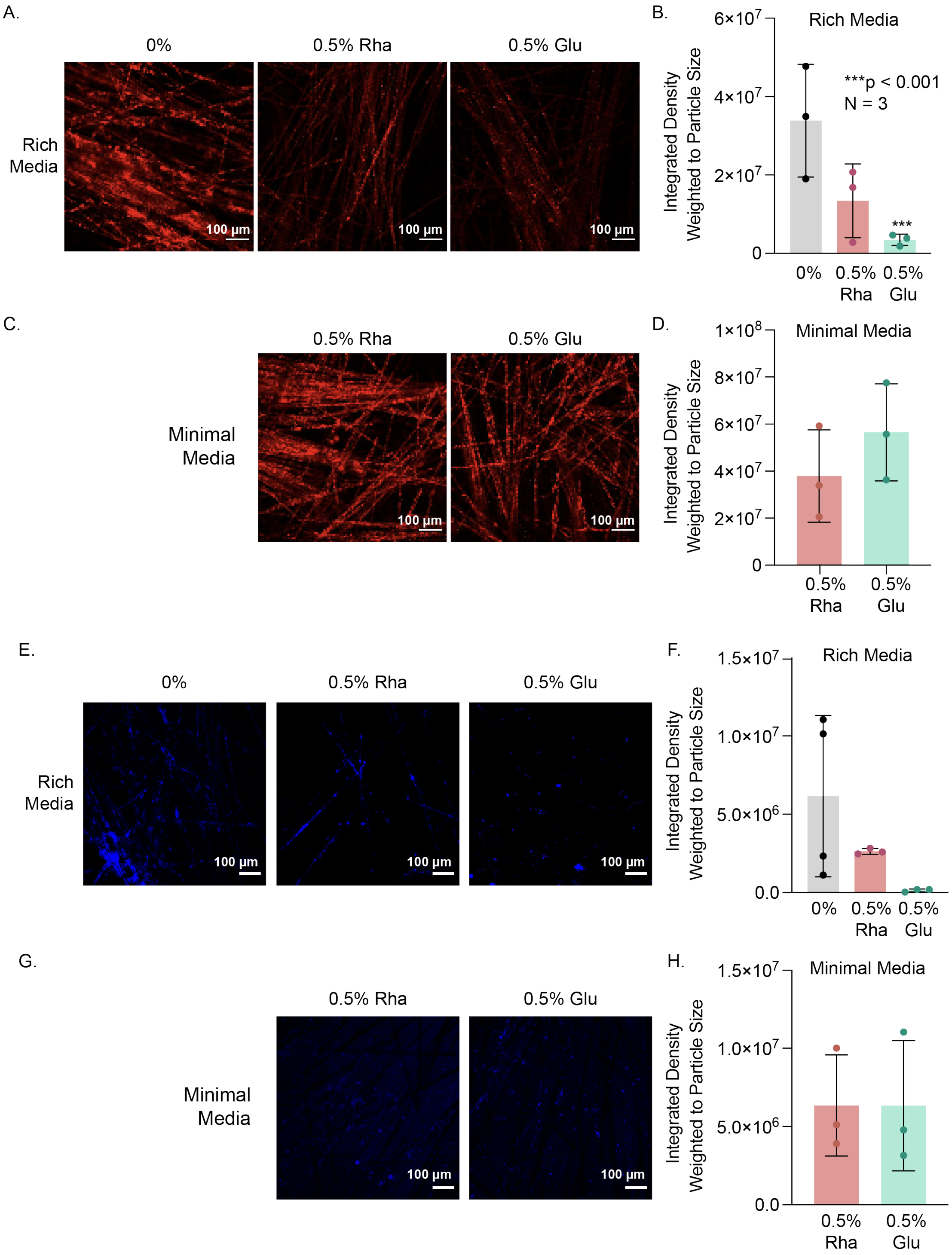

Figure S2. Biomolecule concentrations in the EPS quantified by confocal microscopy. **A-D.** Protein concentration of biofilms grown on glass wool for 48 h at 28 ºC quantified by SyproRuby stain. **E – H.** Carbohydrate concentration was also quantified using confocal microcopy of biofilms grown for 48 h on glass wool at 28 ºC and stained with Calcofluor White. Scale bar represents 100 µm. Statistical significance determined using two-way ANOVA analysis with a post-hoc Tukey test and represents significance between rhamnose and the 0% control in rich media or rhamnose and the 0.5% (*w/w*) control in minimal media at the same culture temperature.

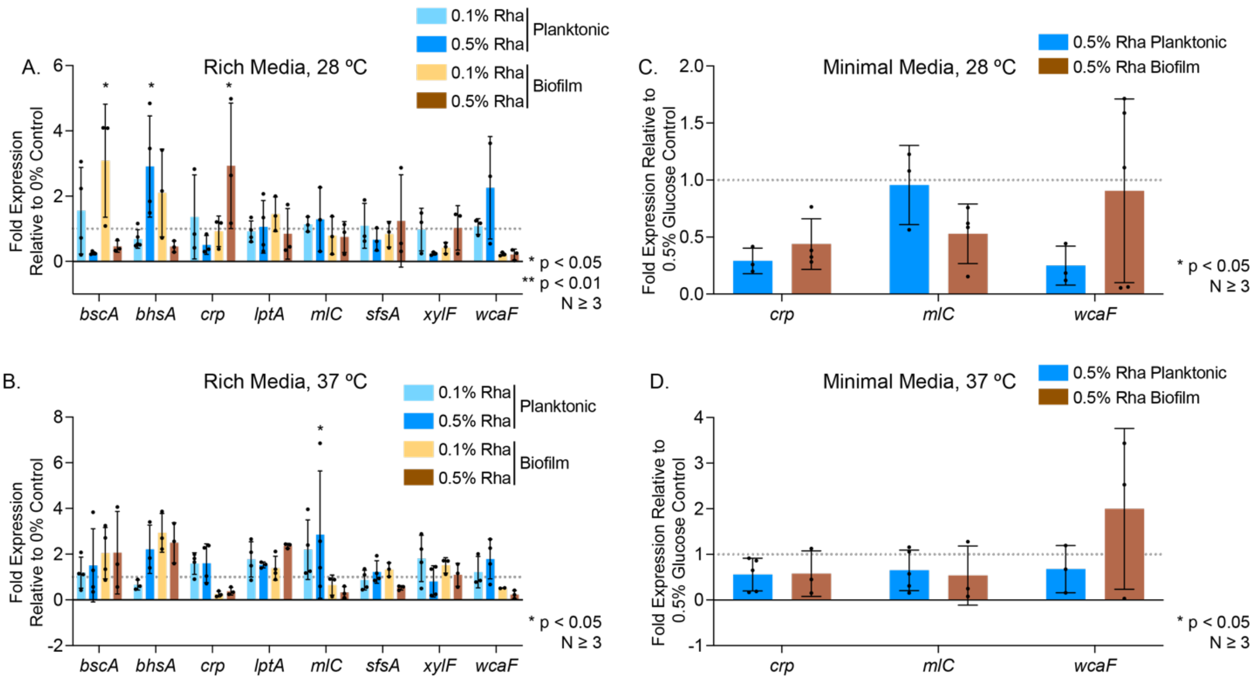

Figure S3. Gene expression data for select transcripts in **A-B** rich and **C-D** and minimal media exposed to varying concentrations of rhamnose. Biofilms were grown on glass wool over 48 h at the temperatures indicated. Planktonic cells were harvested after 24 h growth. Grey dashed line indicates the comparative fold expression = 1 of control (0% sugar for LB/rich media or 0.5% (*w/w*) glucose for M9/minimal media). Statistical significance determined using two-way ANOVA analysis with a post-hoc Tukey test in GraphPad Prism 10.

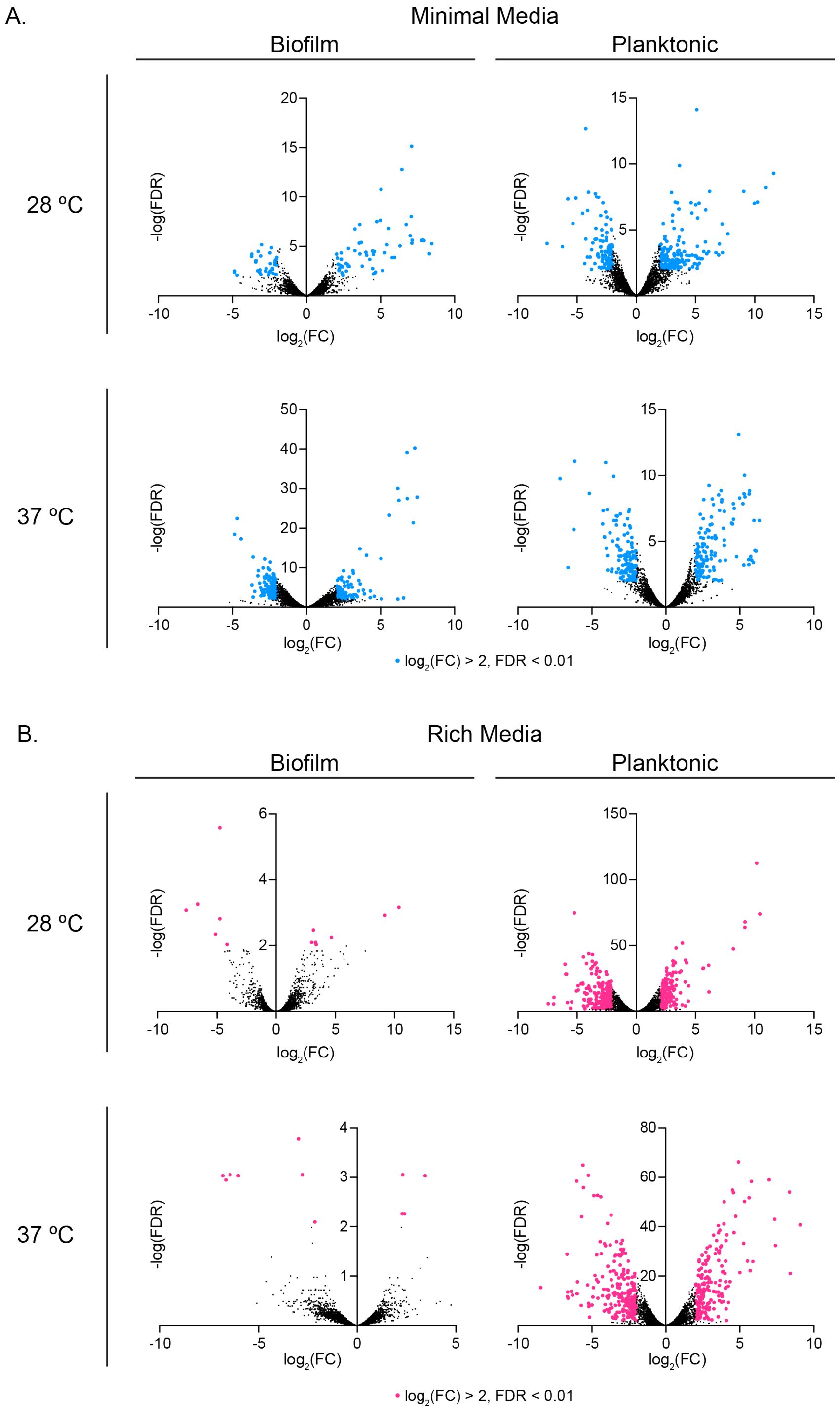

Figure S4. Volcano plots of RNA-seq data analyzed with edgeR for biofilm and planktonic cells harvested from **A.** minimal media (M9) and **B.** rich media (LB). Color data (blue for minimal media, pink for rich media) indicate genes that are deemed "significant" in their differential expression, with $\log_2$(Fold Change) > 2 and false discovery rate < 0.01.

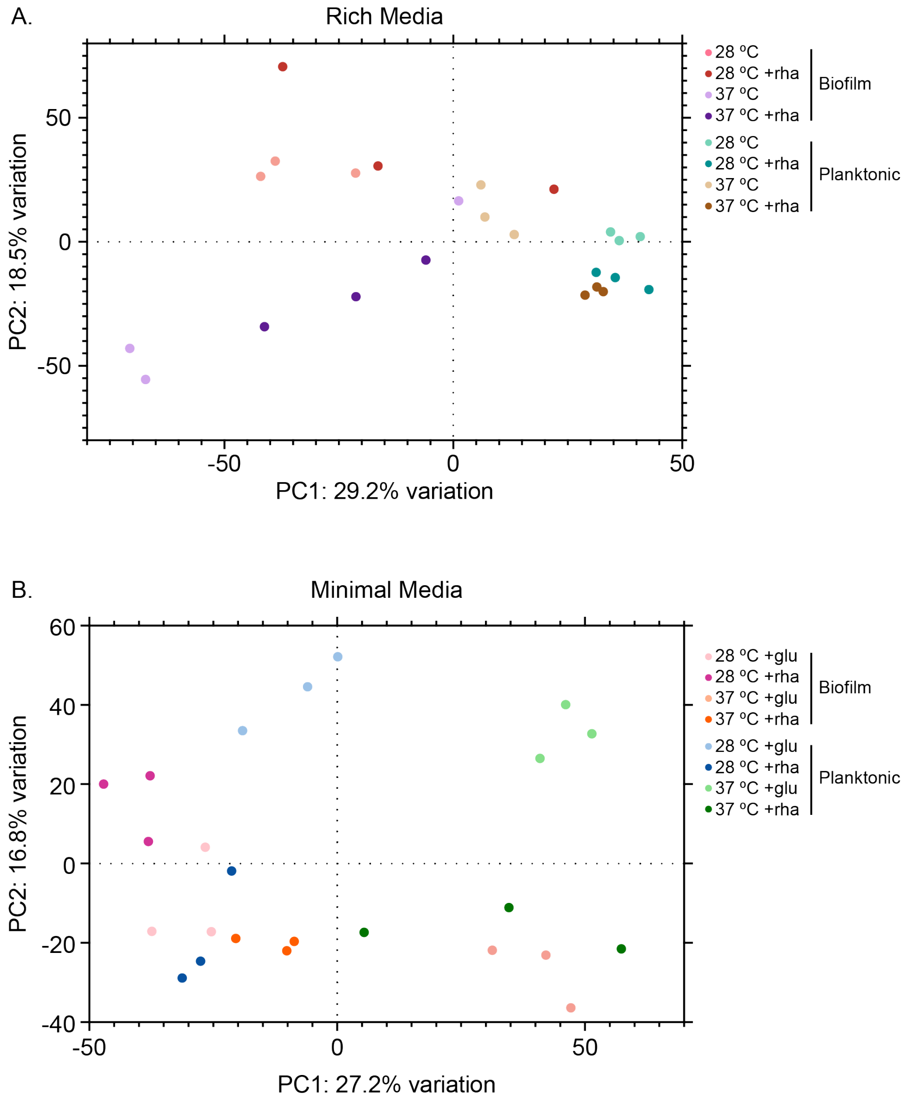

Figure S5. PCA plots of RNA-seq data analyzed with edgeR and limma PCA analysis for biofilm and planktonic cells harvested from **A.** rich media (LB) and **B.** minimal media (M9). Biological triplicates are plotted as the same color, as indicated by the experimental condition legend on the right.

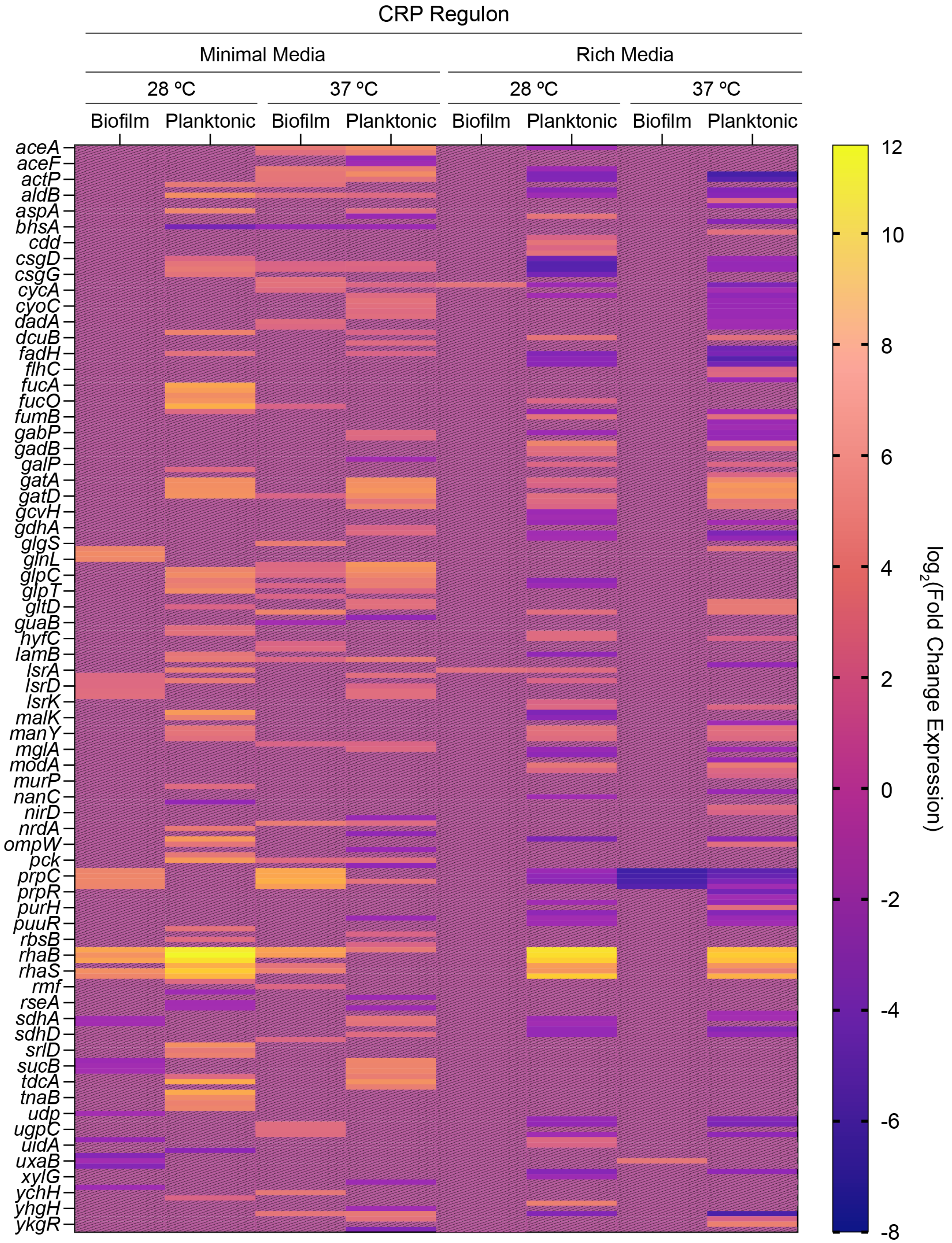

Figure S6. Heatmap of the $\log_2$(Fold Change) expression of the CRP regulon an EcoCyc Omics analysis all growth and media conditions. Hashed and faded cells represent genes that did not have significance as determined by our edgeR differential expression analysis cutoffs of $\log_2$(FC) > 2 and $p < 0.01$.

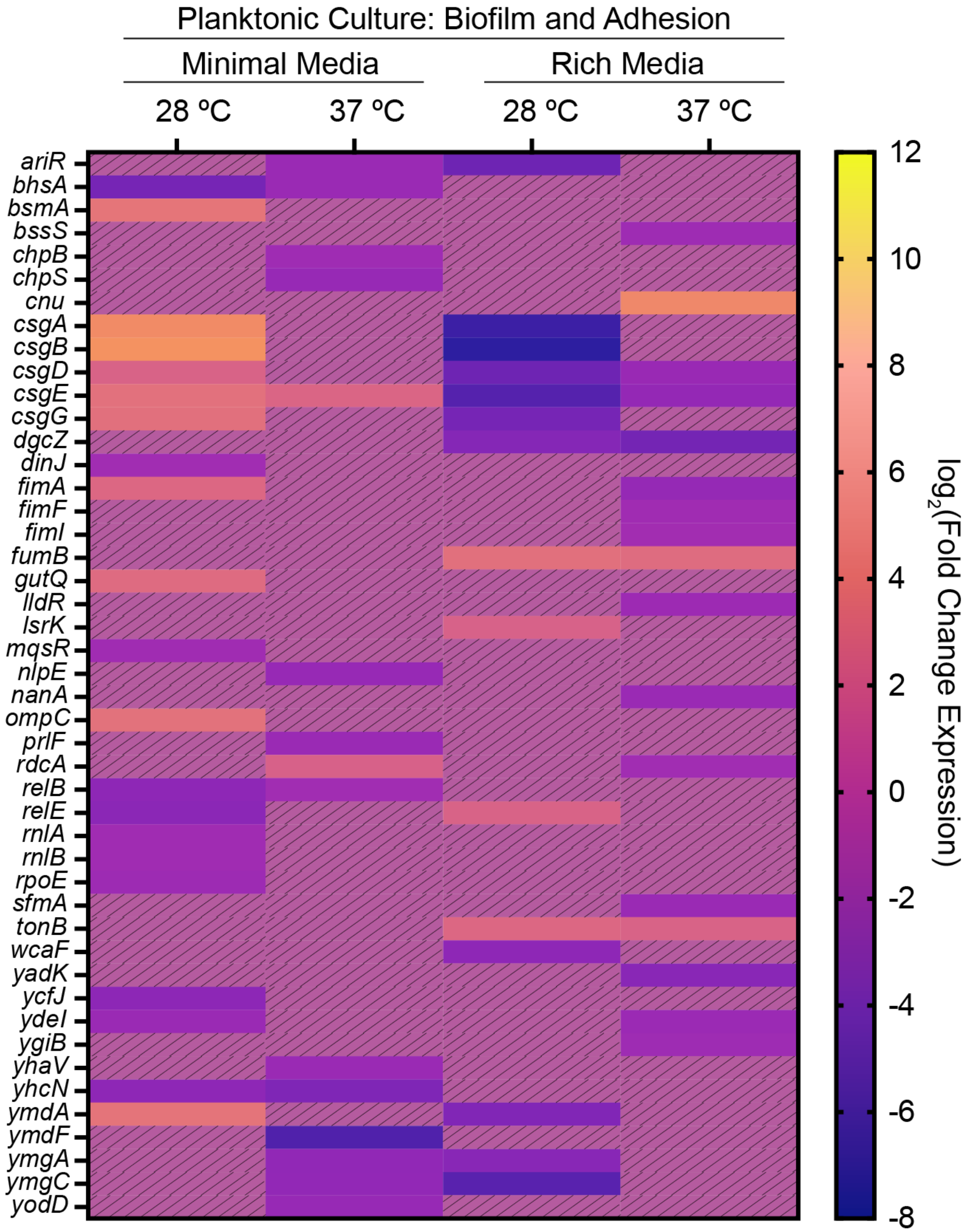

Figure S7. Heatmap of the $\log_2$(Fold Change) expression of biofilm and adhesion transcripts identified in an EcoCyc Omics analysis for planktonic cells at all growth and media conditions. Hashed and faded cells represent genes that did not have significance as determined by our edgeR differential expression analysis cutoffs of $\log_2(FC) > 2$ and $p < 0.01$.
